# The association between soluble CD163, disease severity, and ursodiol treatment in patients with primary biliary cholangitis

**DOI:** 10.1097/HC9.0000000000000068

**Published:** 2023-03-24

**Authors:** Lars Bossen, Tobias Stemann Lau, Mette Bak Nielsen, Marlene Christina Nielsen, Astrid Højmark Andersen, Peter Ott, Sabine Becker, Henning Glerup, Lise Svenningsen, Martin Eivindson, Linda Kornerup, Niels Bjørndal Kjeldsen, Anders Neumann, Holger Jon Møller, Peter Jepsen, Henning Grønbæk

**Affiliations:** 1 Department of Hepatology & Gastroenterology, Aarhus University Hospital, Aarhus, Denmark; 2 Institute of Pathology, Aarhus University Hospital, Aarhus, Denmark; 3 Department of Clinical Biochemistry, Aarhus University Hospital, Aarhus, Denmark; 4 Diagnostic Centre, University Research Clinic for Innovative Patient Pathways, Silkeborg Regional Hospital, Silkeborg, Denmark; 5 Department of Internal Medicine, Horsens Regional Hospital, Horsens, Denmark; 6 Department of Internal Medicine, Herning Regional Hospital, Herning, Denmark; 7 Department of Internal Medicine, Randers Regional Hospital, Randers, Denmark; 8 Department of Internal Medicine, Viborg Regional Hospital, Viborg, Denmark

## Abstract

The macrophage activation marker soluble (s)CD163 is associated with disease severity and prognosis in patients with primary biliary cholangitis (PBC). Ursodeoxycholic acid (UDCA) treatment attenuates fibrosis progression in PBC patients, but its effect on macrophage activation is unclear. We examined the effect of UDCA on macrophage activation, as determined by sCD163 levels.

**Methods:**

We included 2 cohorts of PBC patients; 1 cohort with prevalent PBC patients, and 1 cohort of incident PBC patients before start of UDCA treatment and with follow-up after 4 weeks and 6 months. We measured sCD163 and liver stiffness in both cohorts. Further, we measured sCD163 and TNF-α shedding *in vitro* in monocyte-derived macrophages after UDCA and lipopolysaccharide incubation.

**Results:**

We included 100 patients with prevalent PBC [93% women, median age 63 y (interquartile range: 51–70)] and 47 patients with incident PBC [77% women, median age 60 y (49–67)]. Prevalent PBC patients had a lower median sCD163 of 3.54 mg/L (2.77–4.72) than incident PBC patients with a median sCD163 of 4.33 mg/L (2.83–5.99) at inclusion. Patients with an incomplete response to UDCA and patients with cirrhosis had higher sCD163 than responders to UDCA and noncirrhosis patients. After 4 weeks and 6 months of UDCA treatment median sCD163 decreased by 4.6% and 9.0%, respectively. In *in vitro* experiments, UDCA attenuated shedding of TNF-α, but not sCD163, from monocyte-derived macrophages.

**Conclusion:**

In PBC patients, sCD163 levels correlated with liver disease severity and treatment response to UDCA. Further, after 6 months of UDCA treatment, we observed a decrease in sCD163, which may be related to the treatment.

## Graphical abstract

**Figure:**
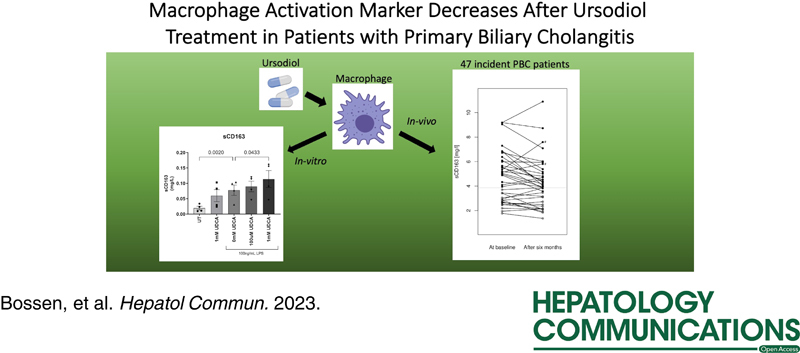

## INTRODUCTION

Primary biliary cholangitis (PBC) is a chronic autoimmune liver disease affecting intrahepatic bile ducts.^1^ Disease progression is very heterogeneous with a substantial part of the patients developing biliary cirrhosis and end-stage liver disease.^2^ Inflammation in PBC is driven by an immune response toward mitochondrial autoantigens located in apoptotic bodies from biliary epithelial cells (BECs) and includes both CD4 and CD8 cells, which activate macrophages through granulocyte macrophage colony-stimulating factor.^3^–^5^ The activated macrophages, together with activated T cells and anti-mitochondrial antibodies (AMAs), produce a proinflammatory response with subsequent damage to BECs resulting in biliary inflammation and portal fibrosis.^6^ Hence, macrophages are suggested to serve as a link between injury mediated by the innate immune system and BEC apoptosis.^7^,^8^ Further, in biopsies from PBC patients, macrophages comprise ~30% of mononuclear cells found in the cellular infiltrate.^9^

The macrophage activation marker soluble CD163 (sCD163)^10^,^11^ is associated with disease severity and prognosis^12^–^20^ and decreases after treatment^13^,^14^,^19^,^21^,^22^ in a number of acute and chronic inflammatory liver diseases. Recently, our group showed that sCD163 was associated with liver disease severity, for example, alkaline phosphatase (ALP) and with long-term risk of liver-related events in PBC patients.^23^ However, before-and-after treatment levels of sCD163 were not investigated, and reported levels of sCD163 were not separated according to the time-point in the disease (“prevalent” or “incident”).

Ursodeoxycholic acid (UDCA) is the first-line drug for the treatment of PBC patients.^2^ UDCA actions include protection of cholangiocytes against toxic effects of bile acids, stimulation of impaired biliary secretion, and beneficial changes of the bile acid pool composition, along with antiapoptotic effects in hepatocytes and cholangiocytes.^7^,^24^ These effects are associated with attenuation of fibrosis progression and improved liver transplantation-free survival. However, it is unknown if UDCA affects macrophage activation, determined by sCD163 levels, in PBC patients.

We aimed to investigate the association between sCD163 and disease severity in prevalent PBC patients, and the effect of UDCA on macrophage activation in incident PBC patients. We hypothesized that sCD163 is associated with liver disease severity and that UDCA treatment reduces macrophage activation, determined by sCD163 levels.

## METHODS

### Participants and study design

From 2016 to 2019 we included PBC patients at our tertiary outpatient clinic into 2 different cohorts. In the first cohort, we included all prevalent PBC patients, that is patients who were currently being treated with UDCA or had previously been treated with UDCA. In the second cohort, we included incident PBC patients before they started treatment with UDCA. Patients included as incident PBC patients were seen again after 4 weeks and 6 months after UDCA treatment initiation. We included patients from both our tertiary center and from all regional hospitals in the Central Denmark Region, who referred patients to our clinic for inclusion into the study cohorts. PBC diagnosis followed current guidelines from the European Association for the Study of the Liver (EASL) and American Association for the Study of Liver Diseases (AASLD),^2^,^25^ and autoimmune hepatitis (AIH) overlap diagnosis followed current guidelines from AASLD.^25^ For PBC 2 of the 3 criteria were fulfilled: (1) elevated ALP, (2) presence of AMAs, and (3) a liver biopsy specimen showing florid bile duct lesions. For the AIH diagnosis, the patients had (1) elevated alanine aminotransferase, (2) elevated serum IgG and/or presence of smooth muscle antibodies, and (3) a liver biopsy with moderate to severe interface hepatitis.

The studies were approved by the local ethics committee and reported to clinicaltrials.gov (NCT02924701 and NCT02931513). The studies were conducted in accordance with both the Declarations of Helsinki and Istanbul, and all participants gave written consent before inclusion.

### Data collection

On the day of inclusion, we collected data on age, gender, date of diagnosis, liver biopsy, UDCA treatment, and AIH overlap. In addition, we performed transient elastography (TE) to measure liver stiffness using a FibroScan (M or XL probe as necessary), and collected blood samples. In the incident PBC patients, TE and blood sampling were also done at the 4-week and 6-month visits. Alanine aminotransferase, bilirubin, ALP, platelets, IgM, IgG, INR, albumin, and creatinine were measured in blood samples.

We measured sCD163 as a standard biochemical test by an in-house sandwich ELISA using a BEP-2000 ELISA-analyzer (Dade Behring)^26^ with a reference interval of 0.69–3.86 mg/L.^10^ The intraindividual variation of sCD163 is 9.0% and the interindividual variation ~35%.^27^ In the cohort of patients with prevalent PBC, we created a dichotomous variable of cirrhosis being “yes” if the patients fulfilled one or more of the following criteria: (1) cirrhosis on a previous liver biopsy, (2) history of variceal bleeding or ascites or varices at the latest performed gastroscopy, (3) liver stiffness ≥16.9 kPa at inclusion,^28^ and otherwise “no.” Moreover, we used the cutoff presented by Cristoferi et al^29^ to distinguish between those with high risk of advanced fibrosis (TE >11 kPa) and those with low risk (TE <11 kPa), and the new Baveno VII cutoffs for compensated advanced chronic liver disease, where a TE between 10 and 15 kPa is suggestive of advanced fibrosis and a TE above 15 kPa is highly suggestive of advanced fibrosis.^30^ Further, in the cohort with prevalent PBC patients, patients with ALP>1.67× the upper limit of normal (175 U/I) or abnormal bilirubin (>25 µmol/L) after at least 1 year of UDCA treatment were considered incomplete responders to UDCA treatment as defined by the POISE criteria.^31^ All biopsies in the cohort of incident PBC patients were staged according to the Ludwig system.^32^ Further, in 35 biopsies with enough material available, we used immunohistochemistry to stain and digital image analysis to quantify CD163 around the portal tracts. For further details, see Supplemental Methods (http://links.lww.com/HC9/A164).

### UDCA effect on macrophage shedding of sCD163

The ADAM17 enzyme is responsible for the shedding of sCD163 and TNF-α from macrophages,^33^ and UDCA has been shown to inhibit the ADAM17-driven shedding of TNF-α.^34^ We therefore hypothesized that UDCA inhibits the ADAM17-driven shedding of sCD163 too. To evaluate this *in vitro*, we measured sCD163 and TNF-α shedding from monocyte-derived macrophages after UDCA and lipopolysaccharide (LPS) incubation. For further details, see Supplemental Methods (http://links.lww.com/HC9/A164). To evaluate the hypothesis *in vivo*, we measured concurrent levels of sCD163 and TNF-α before and after UDCA treatment. Together, these analyses will help us understand whether UDCA reduces inflammation, and whether such an effect involves macrophages or it involves activated T cells, which shed TNF-α but not sCD163.^2^ TNF-α was measured as part of a V-PLEX proinflammatory kit also analyzing interferon-γ, IL-1β, IL-2, IL-4, IL-6, IL-8, IL-10, IL-12p70, and IL-13.

### Statistical analysis

Patient characteristics from both cohorts are reported as percentage and mean (SD) if normally distributed and median (interquartile range, IQR) if non-normally distributed. Correlation analyses were performed using the Spearman rank correlation. In the cohort of incident PBC patients, 46 patients attended the 4-week visit of whom 43 had their sCD163 measured, and 37 patients attended the 6-month visit. For repeated measurements, a 1-way repeated ANOVA was used. There were a few missing data on TE in the cohort with incident patients, either because it was impossible to obtain 10 valid measurements or because the patient did not meet all criteria for the examination (eg, <4 h fasting or pacemaker).

## RESULTS

### Baseline characteristics

We included 100 prevalent PBC patients of whom 93 (93%) were women and the median age was 63 years (IQR: 51–70). Median time from diagnosis to inclusion was 6.6 years (IQR: 2.5–13.2). The patients had a median sCD163 of 3.54 mg/L (IQR: 2.77–4.72) and a median TE of 5.8 kPa (IQR: 4.8–8.4), and 8 patients had a TE >16.9 kPa indicating cirrhosis (Table 1).

**TABLE 1:** Patient characteristics at inclusion

	Prevalent patients	Incident patients
N	100	47
Age, median (IQR) (y)	62.5 (51–70)	60 (49–67)
Female, n (%)	93 (93)	36 (76.6)
Time since diagnosis, y (IQR)	6.61 (2.48–13.17)	—
AMA positive at diagnosis, n (%)	72 (75.8)	41 (87.2)
UDCA treatment, n (%)	95 (95)	43 (91.5)
AIH overlap, n (%)	25 (25)	3 (6.4)
ALP, median (IQR), U/I	140 (107–213)	281 (170–362)
ALT, median (IQR), U/I	33 (23–48)	72 (40–109)
Bilirubin, median (IQR), µmol/L	9 (6–12)	10 (7–13)
Albumin, median (IQR), g/L	37 (36–39)	37 (35–40)
Platelets, median (IQR), 10^9^/L	245 (189–296)	257 (216–320)
Coagulation factor 2, 7, and 10, median (IQR)	1.06 (0.92–1.21)	1.04 (0.86–1.18)
IgM, median (IQR), g/L	2.68 (1.49–3.82)	2.95 (1.87–4.07)
TE stiffness, median (IQR), kPa	5.8 (4.8–8.4)	6.2 (4.7–8.7)
sCD163, median (IQR), mg/L	3.54 (2.77–4.72)	4.33 (2.83–5.99)

Abbreviations: AIH, autoimmune hepatitis; ALP, alkaline phosphatase; ALT, alanine aminotransferase; AMA, anti-mitochondrial antibodies; IQR, interquartile range; sCD163, soluble CD163; TE, transient elastography; UDCA, ursodeoxycholic acid.

Among the 47 incident PBC patients, 36 (76.6%) were women and the median age was 60 years (IQR: 49–67). At inclusion, the median sCD163 was 4.33 mg/L (IQR: 2.83–5.99) and the median TE was 6.2 kPa (IQR: 4.7–8.7) (Table 1). Forty-one of the 47 patients underwent liver biopsy of whom 26 (63.4%) had stage 1 fibrosis according to the Ludwig system; 10 (24.4%) had stage 2; 2 (4.9%) had stage 3; and 3 (7.3%) had stage 4.

### Association between sCD163 and disease severity

In the cohort of prevalent PBC patients, those with cirrhosis (n=13) had higher median sCD163 levels (5.39 mg/L, IQR: 3.98–5.67) than those without cirrhosis (n=87, median=3.21 mg/L, IQR: 2.67–4.47), *p*<0.001. Similarly, those with higher risk of advanced fibrosis had higher median sCD163 (n=18, median=5.3 mg/L, IQR: 4.4–6.4) than those with lower risk of advanced fibrosis (n=82, median=3.2 mg/L, IQR: 2.7–4.4), *p*<0.001. Further, those who was highly suggestive of compensated advanced chronic liver disease had higher median sCD163 (n=9, median=5.6 mg/L, IQR: 5.4–6.6) than those who was suggestive of compensated advanced chronic liver disease (n=10, median=4.7 mg/L, IQR: 3.7–5.4), who had higher median sCD163 than those with low risk of compensated advanced chronic liver disease (n=81, median=3.2 mg/L, IQR: 2.7–4.3), *p*<0.001. The 26 incomplete responders to UDCA had higher median sCD163 (4.51 mg/L, IQR: 3.10–5.50) than the 64 responders (median=3.16 mg/L, IQR: 2.58–4.20), *p*=0.004. Ten patients had been treated with UDCA for <1 year. There was no difference in disease duration or the proportion of patients with cirrhosis between responders and incomplete responders.

**FIGURE 1:**
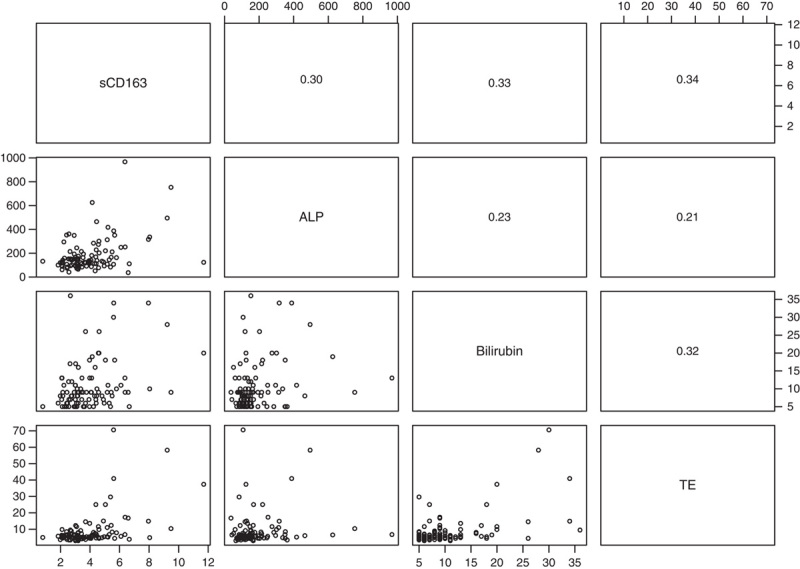
Scatterplots (lower left panels) and Spearman rho correlations (upper right panels) between sCD163, ALP, bilirubin, and TE in 100 prevalent PBC patients. Notes: sCD163 (mg/L), ALP (U/I), bilirubin (µmol/L), TE (kPa). Abbreviations: ALP, alkaline phosphatase; PBC, primary biliary cholangitis; sCD163, soluble CD163; TE, transient elastography.

**FIGURE 2:**
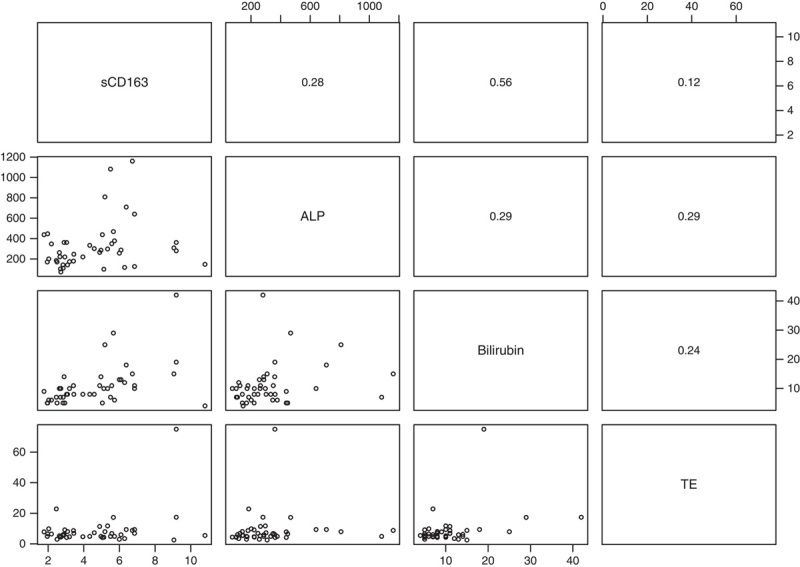
Scatterplots (lower left panels) and Spearman rho correlations (upper right panels) between sCD163, ALP, bilirubin, and TE in 44 incident PBC patients at baseline (3 patients had missing data on TE). Notes: sCD163 (mg/L), ALP (U/I), bilirubin (µmol/L), TE (kPa). Abbreviations: ALP, alkaline phosphatase; PBC, primary biliary cholangitis; sCD163, soluble CD163; TE, transient elastography.

sCD163 correlated with ALP and bilirubin in both the prevalent and incident PBC patients, but the correlation strength was modest (Figures 1 and 2). Further, sCD163 and TE correlated in the prevalent PBC patients (Figure 1). Correlations between sCD163 and bilirubin, TE, and ALP were stronger in the 26 incomplete responders than in the 64 responders.

We did not observe any correlation between sCD163 and CD163 around the portal tracts in 35 biopsies (Spearman rho=0.24, *p*=0.162). In 32 patients with <3 months between liver biopsy and blood sampling, the correlation between sCD163 and CD163 around the portal tracts was considerably stronger (Spearman rho=0.39, *p*=0.025).

### UDCA and sCD163

**FIGURE 3:**
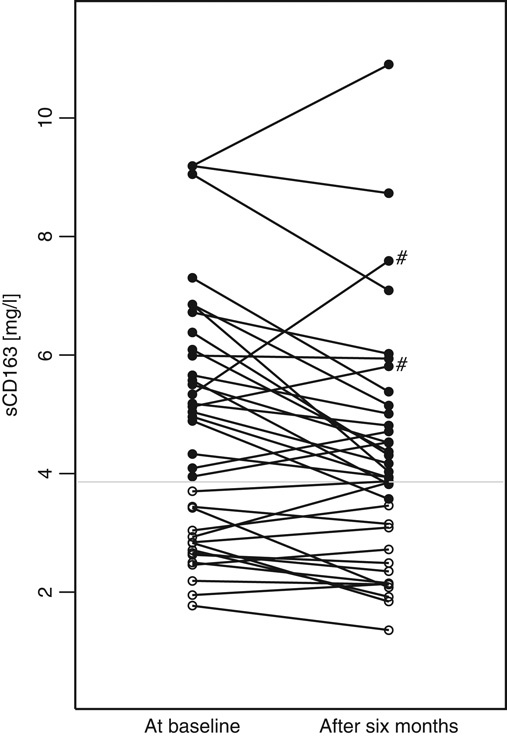
Changes in sCD163 after 6 months of UDCA treatment. Open circles had normal sCD163 at baseline and filled circles had sCD163>3.86 mg/L (ULN) at baseline. Gray horizontal line at sCD163=3.86 mg/L. The “#” marks 2 patients whose ALP increased from baseline to 6 months. Note: sCD163 (mg/L). Abbreviations: ALP, alkaline phosphatase; sCD163, soluble CD163; UDCA, ursodeoxycholic acid; ULN, upper limit of normal.

Following UDCA treatment, median sCD163 decreased by 4.6% after 4 weeks, and by 9.0% after 6 months (Table 2). Of the 37 patients with follow-up data after 6 months of UDCA treatment, 26 had a decrease in sCD163 and 11 had an increase in sCD163 (Figure 3). Of those 11, 6 had normal sCD163 at baseline; and of those 6, 5 had a sCD163 still within the normal range after 6 months and the 1 remaining patient had the sCD163 increased to 3.87 mg/L (Figure 3). Two of the 11 also had an increase in their ALP (Figure 3).

**TABLE 2:** Median (IQR) levels of sCD163, ALP, TE, and TNF-α at inclusion and after 4 weeks and 6 months of UDCA treatment

	Inclusion	4 weeks	6 months	*p*
sCD163, mg/L	4.33 (2.83–5.99)	4.13 (2.7–5.44)	3.94 (2.61–5.08)	0.007
ALP, U/I	281 (170–362)	175 (122–209)	155 (128–185)	<0.001
TE stiffness, kPa	6.2 (4.7–8.7)	6.3 (4.8–8.5)	6.7 (4.6–8.7)	0.825
TNF-α, pg/mL	1.83 (1.41–2.36)	1.76 (1.40–2.40)	1.60 (1.23–2.26)	0.052

Notes: Medians are reported for all patients with available data at the different time points.

*p*-Values are from one-way repeated ANOVA analyses after transformation of the variables using the natural logarithm.

Abbreviations: ALP, alkaline phosphatase; IQR, interquartile range; sCD163, soluble CD163; TE, transient elastography; UDCA, ursodeoxycholic acid.

**FIGURE 4:**
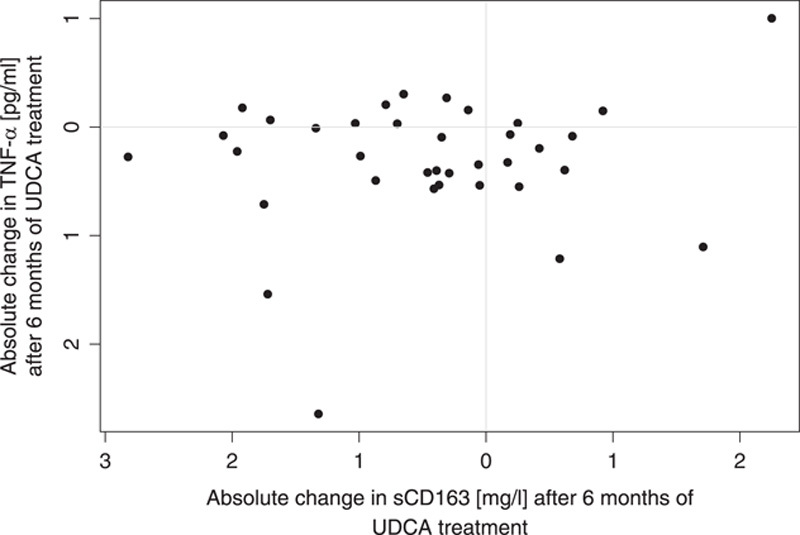
Changes in sCD163 and TNF-α after 6 months of UDCA treatment. Notes: sCD163 (mg/L); TNF-α (pg/mL). Abbreviations: sCD163, soluble CD163; UDCA, ursodeoxycholic acid.

Before UDCA treatment, sCD163 and TNF-α correlated moderately (Spearman rho=0.38, *p*=0.008). After 6 months of treatment with UDCA, TNF-α was lower than that before treatment (Table 2). However, changes in sCD163 and TNF-α after UDCA treatment did not correlate and 16 of the 36 patients had discordant changes in sCD163 and TNF-α (Figure 4). Results from the remaining inflammatory markers are given in Supplementary Table 1 (http://links.lww.com/HC9/A164).

### UDCA and macrophage shedding of sCD163 and TNF-α

**FIGURE 5:**
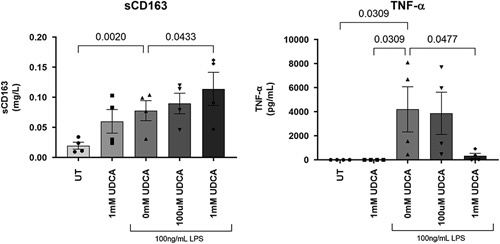
*In vitro* analysis of sCD163 and TNF-α shedding from monocyte-derived macrophages preincubated with UDCA for 1 hour followed by incubation with 100 ng/mL LPS for 1 hour. Numbers above brackets are *p* values, n=4. Notes: sCD163 (mg/L), TNF-α (pg/mL). Abbreviations: LPS, lipopolysaccharide; sCD163, soluble CD163; UDCA, ursodeoxycholic acid.

In *in vitro* analysis, LPS stimulated sCD163 shedding from macrophages as expected (Figure 5). Further, there was a tendency to a higher sCD163 shedding with increasing UDCA concentration both in those with and those without LPS incubation. There was no TNF-α shedding in those not stimulated with LPS. In those stimulated with LPS, the highest dose of UDCA (1 mM) decreased the TNF-α shedding to a level almost comparable to that observed in those without LPS stimulation (Figure 5).

To summarize the changes in sCD163 and TNF-α from the *in vivo* and *in vitro* analyses, there was a reduction in median sCD163 and TNF-α after UDCA treatment *in vivo*, but only shedding of TNF-α from macrophages decreased, and only at the highest concentration of UDCA, in the *in vitro* experiment.

## DISCUSSION

Among patients with an established diagnosis of PBC, we confirmed that sCD163 is a marker of liver disease severity with increased levels in PBC patients with cirrhosis and in patients with an incomplete response to UDCA. As novel findings, in a prospective cohort of incident PBC patients, our data suggest that UDCA treatment reduced macrophage activation as determined by sCD163 levels. This finding may suggest that UDCA have an anti-inflammatory effect partly mediated through the inhibition of macrophage activation.

Levels of sCD163 presented here are comparable with those observed in early stage disease of other chronic liver diseases, that is hepatitis B and C and alcohol-associated liver disease,^12^,^35^,^36^ and levels in the group of “prevalent” PBC patients are comparable to those previously presented in PBC patients.^23^,^37^ Further, we observed that patients with an incomplete response had higher levels of sCD163 than those with a complete response similar to what was previously reported.^23^ Unfortunately, no data describing the levels of sCD163 before and after UDCA treatment have previously been published in other cholestatic liver diseases such as PSC, and hence we have no such data to compare with.

In PBC pathogenesis, liver macrophages are activated by CD4 and CD8 lymphocytes stimulated by the pyruvate dehydrogenase complex (PDC-E2), which is the autoantigen targeted by AMAs.^3^–^5^ The activated macrophages produce a proinflammatory response through the activation of cytotoxic T cells as well as Th1-positive and Th17-positive T cells producing interferon-γ and TNF-α. This proinflammatory milieu is associated with subsequent damage to BECs and destruction of bile ducts accompanied by bile leaking into the liver parenchyma causing damage to hepatocytes with subsequent biliary inflammation and portal fibrosis.^2^,^6^ In line with this, we observed an association between sCD163 and TNF-α in the newly diagnosed PBC patients before UDCA treatment. Further, when macrophages are activated, they may contribute to fibrogenesis, and it has consistently been shown that sCD163 levels are associated with fibrosis in other chronic inflammatory liver diseases.^38^ Thus, macrophages are thought to play a key role in PBC pathogenesis with inflammation and later fibrosis development.

We showed that UDCA has an anti-inflammatory effect with reduced levels of macrophage activation marker sCD163 and TNF-α after 6 months of treatment. The anti-inflammatory effect of UDCA has previously been reviewed,^24^ and in short, UDCA is reported to decrease TNF-α, TGF-β, IL-2, IL-4, and INF-γ, whereas IL-1, IL-6, IL-8, and IL-12 are unaffected by UDCA treatment.^39^–^41^ In our *in vitro* experiment, there was no reduction in sCD163 shedding from monocyte-derived macrophages after incubation with UDCA, whereas there was a large reduction of TNF-α shedding in those incubated with high doses of UDCA. This suggests that the anti-inflammatory effect on the macrophages observed *in vivo* is indirect. Further, our observation of no correlation between changes in sCD163 and TNF-α suggest that UDCA does not inhibit macrophages and activated T cells *per se*. Thus, the decrease in both sCD163 and TNF-α after UDCA treatment may suggest that both macrophages and T-cells are affected by UDCA. The discrepancy between sCD163 and TNF-α changes may also be due to different half-lives, as shown after LPS stimulation.^42^ Previous studies investigating sCD163 and TNF-α changes showed conflicting results: Two studies found parallel increases after induction of inflammation,^43^,^44^ whereas one study found unparallel changes after lifestyle interventions in obese children.^13^ In our study, patients with high sCD163 before start of treatment demonstrated a consistent decrease in sCD163 levels during treatment, whereas this was not the case in patients with sCD163 levels within the normal range before treatment. This possibly reflects that patients with normal sCD163 levels have less inflammation in the liver related to PBC, and hence UDCA is less likely to reduce the inflammation.

Currently, there is a clinical focus on identifying noninvasive markers of disease progression, prognosis, and treatment response in PBC research.^45^,^46^ This is the first study to present repeated measurements of the macrophage activation marker sCD163 before and after UDCA treatment, and to show that the marker decreases after UDCA treatment. Moreover, we observed that the levels of sCD163 were higher in the cohort of incident PBC patients than in patients with prevalent PBC, suggesting that the effect of UDCA treatment on macrophage activation is long lasting. It is of key interest to identify patients with incomplete response to UDCA, preferably already at diagnosis.^47^ In this study, we observed that patients who had an incomplete response to UDCA had higher sCD163 levels than responders. This finding indicates a possible role for the prediction of such patients using sCD163, and we suggest future trials and long-term observational studies to include sCD163 to further investigate the role of macrophage activation in PBC patients.

The strength of the present study is the high number of well-characterized PBC patients including follow-up in a smaller group of newly diagnosed patients before and after UDCA treatment. However, it is a limitation, that we only have 6 months’ follow-up, which limits our ability to investigate response to UDCA and whether pretreatment sCD163 levels predict response to UDCA. Moreover, we found relatively small changes in sCD163 after UDCA treatment, in relation to the natural biological variation, but even though this may suggest that smaller changes are not detected if analyzed several times in the same patient we did observe a significant change in the group of patients investigated.

In conclusion, we demonstrated an association between disease severity and macrophage activation in patients with PBC, and that incomplete responders to UDCA had higher sCD163 levels than responders. Further, our data may suggest that UDCA treatment indirectly reduced macrophage activation in PBC patients as determined by sCD163 levels.

## Supplementary Material
