# Supplementary material for: The association between soluble CD163, disease severity, and ursodiol treatment in patients with primary biliary cholangitis
Source: Hepatol Commun. 2023 Mar 24;7(4):e0068. doi: 10.1097/HC9.0000000000000068 (PMC10043550; doi:10.1097/HC9.0000000000000068)
Supplement: Supplementary file 1 [file hc9-7-e0068-s001.docx]

**Supplementary material for “Ursodeoxycholic acid attenuates macrophage activation in patients with primary biliary cholangitis”**

**Supplementary methods**

*Immunohistochemistry*

Four-μm FFPE tissue sections were immunohistochemically stained on the Ventana Benchmark Ultra automated slide stainer (Ventana Medical Systems, Roche, Oro Valley, AZ) using standard methods. Slides were pretreated for 32 minutes in CC1 and incubated for 20 minutes using primary monoclonal mouse anti-human CD163 RTU (catalogue no. 760-4437, clone MRQ-26, Ventana Medical Systems, Roche). Primary antibody detection was performed using the OptiView DAB detection kit (Ventana Medical Systems, Roche). Sections of appendix, tonsil, liver and pancreas were included on each slide as positive/negative controls.

*Digital Image Analysis*

Images of stained tissue sections were digitized at a magnification of x20 using the Hamamatsu Nanozoomer 2.0HT scanner (Hamamatsu Photonics, Hamamatsu City, Japan).

Regions of Interest (ROI) were identified in each image using a Deep Learning based digital image analysis protocol (Visiopharm Integrator System 2020.8, Visiopharm, Hørsholm, Denmark). All identified ROIs were reviewed by a pathologist (MBN) and manually edited where needed.

Expression of CD163 was digitally quantified using VIS (Visiopharm Integrator System, Visiopharm). An area fraction (AF) of immunohistochemical (IHC) positivity was computed as the IHC stained area within the ROIs normalized to the total ROI area in the biopsy.

*In-vitro macrophage shedding of sCD163*

Monocyte-derived macrophages (MDMs) were obtained by maturation of monocytes isolated from healthy blood donors as described in ^1^. In short, buffy coats from 4 donors were obtained from the blood bank at the Department of Clinical Immunology, Aarhus University Hospital, Denmark (project no. 0094). According to Danish law, the use of anonymized buffy coats does not require specific ethical approval. Peripheral blood mononuclear cells (PBMCs) were isolated using density gradient centrifugation on a Histopaque-1077 gradient (Sigma-Aldrich, Munich, Germany). After PBMC isolation, monocytes were isolated using EasySep™ Human Monocyte Isolation Kit (Stemcell Technologies, Vancouver, Canada) according to the manufacturer’s protocol. Monocytes were subsequently cultured in non-treated T-75 flasks in complete maturation media (RPMI-1640 (ThermoFisher Scientific, Waltham, MA) with 10 % FCS, (ThermoFisher Scientific), 100 U /100 µg/mL penicillin/streptomycin (ThermoFisher Scientific), 10 ng/mL macrophage colony-stimulating factor (M-CSF) (Peprotech, Stockholm, Sweden), and 1 ng/mL granulocyte-macrophage colony-stimulating factor (GM-CSF) (Peprotech) for 6 days for differentiation to MDMs. Media were changed every 2-3 days.

After maturation, MDMs were harvested as described in ^1^ and re-seeded in 12-well plates at 1x10^6^ cells/mL. The MDMs were then pre-incubated with either 100µM or 1mM of UDCA^2-4^ (Sigma-Aldrich) for one hour followed by incubation with LPS (Sigma-Aldrich) (100ng/ml) for one hour, after which media was collected and stored at -80°C for later ELISA CD163 analysis. Further, we included control MDMs without UDCA incubation and with and without LPS incubation. At last, the concentration of sCD163 and TNF-α were measured in the supernatant.

**Supplementary table 1:** Median (IQR) levels of IFN-γ, IL-4, IL-6, IL-8, IL-10, and IL-12p70 at inclusion and after 4 weeks and 6 months of UDCA treatment. Medians are reported for all patients with available data at the different time points. Concentrations of IL-1β, IL-2 and IL-13 were below the detection threshold.

|  | **Inclusion** | **4 weeks** | **6 months** |
| --- | --- | --- | --- |
| IFN-γ pg/ml | 14.0 (8.89-20.3) | 10.7 (6.52-16.0) | 11.5 (7.53-15.3) |
| IL-4 pg/ml | 0.018 (0.005-0.034) | 0.021 (0.012-0.027) | 0.015 (0.007-0.026) |
| IL-6 pg/ml | 1.42 (0.76-2.31) | 1.42 (0.93-2.62) | 1.68 (1.09-2.34) |
| IL-8 pg/ml | 25.1 (15.7-37.9) | 22.1 (15.7-33.6) | 17.6 (13.8-29.1) |
| IL-10 pg/ml | 0.65 (0.43-1.00) | 0.61 (0.42-0.79) | 0.52 (0.39-0.64) |
| IL-12p70 pg/ml | 0.21 (0.12-0.40) | 0.23 (0.09-0.48) | 0.17 (0.07-0.52) |

**References**

1. Nielsen MC, Andersen MN, Moller HJ. Monocyte isolation techniques significantly impact the phenotype of both isolated monocytes and derived macrophages in vitro. *Immunology.* 2020;159(1):63-74.

2. Ko WK, Lee SH, Kim SJ, et al. Anti-inflammatory effects of ursodeoxycholic acid by lipopolysaccharide-stimulated inflammatory responses in RAW 264.7 macrophages. *PLoS One.* 2017;12(6):e0180673.

3. O'Dwyer AM, Lajczak NK, Keyes JA, Ward JB, Greene CM, Keely SJ. Ursodeoxycholic acid inhibits TNFalpha-induced IL-8 release from monocytes. *Am J Physiol Gastrointest Liver Physiol.* 2016;311(2):G334-341.

4. Horvatova A, Utaipan T, Otto AC, et al. Ursodeoxycholyl lysophosphatidylethanolamide negatively regulates TLR-mediated lipopolysaccharide response in human THP-1-derived macrophages. *Eur J Pharmacol.* 2018;825:63-74.
